# Prevalence and factors associated with body dysmorphic disorder in women under dermatological care at a Brazilian public institution^☆^^☆☆^

**DOI:** 10.1016/j.abd.2020.06.003

**Published:** 2020-11-18

**Authors:** Mariana Mathias Morita, Maira Renata Merlotto, Cássia Lopes Dantas, Fernando Henrique Olivetti, Hélio Amante Miot

**Affiliations:** Faculty of Medicine, Universidade Estadual Paulista, Botucatu, SP, Brazil

**Keywords:** Alcoholism, Body image, Child abuse, sexual, Depression, Health surveys

## Abstract

**Background:**

Body dysmorphic disorder consists of excessive concern with minimal appearance defects, which causes functional impairment. Its prevalence is estimated to range from 5% to 35% of dermatological patients, especially adult women with esthetic complaints.

**Objectives:**

To investigate the prevalence and factors associated with dysmorphic disorder in female dermatological patients, in a public institution in Brazil.

**Methods:**

This was a cross-sectional study involving adult women attended at a public dermatological service in Brazil. Participants underwent a demographic survey, in addition to a screening form for body dysmorphic disorder (Body Dysmorphic Disorder Examination [BDDE]). The presence of dysmorphic disorder (BDDE > 66 points) was assessed among the participants according to demographic covariates and psychological problems, through logistic regression.

**Results:**

A total of 223 women were evaluated. The BDDE showed high internal consistency (Cronbach’s α = 0.90). It is noteworthy the high prevalence of psychological problems and the fact that over one-third (38%) of the sample presented a high degree of dissatisfaction with their image. The prevalence of dysmorphic disorder was 48% among women with esthetic complaints and 30% among the others (p < 0.01). Lower family income (OR = 2.97), history of domestic violence (OR = 3.23), search for dermatological care due to an esthetic complaint (OR = 2.05), and suicidal ideation (OR = 4.22) were independently associated with the occurrence of body dysmorphic disorder.

**Study limitations:**

This was a single-center study of a non-randomized sample from public service.

**Conclusions:**

Body dysmorphic disorder is prevalent among female dermatological patients; it is associated with traumatic psychological experiences, lower income, affective disorders, and demand for esthetic care. It is essential to recognize the diagnosis in order to treat such patients and refer them for appropriate psychiatric treatment instead of trying to satisfy their esthetic demands.

## Introduction

Body dysmorphic disorder (BDD), or dysmorphophobia, is defined as an excessive and pathological concern with minimal or nonexistent defects in appearance. BDD differs from normal concerns because it causes important functional impairment to the individuals, decreasing their quality of life, distancing them from daily activities, and taking up a lot of their time. Examples of BDD include anorexia, concern with muscle definition, and aspects related to esthetic complaints, such as open pores, facial asymmetry, wrinkles, and discreet spots.^1^, ^2^, ^3^

BDD is quite prevalent among plastic surgery and dermatology patients. It is estimated to affect between 4.5% to 35.2% of dermatological patients, *vs*. 0.7% to 2.3% of the control population. A higher prevalence is observed among adult women (76% of cases) (25 − 40 years) and patients who report esthetic complaints.^4^, ^5^, ^6^, ^7^, ^8^ These seek care in search of successive treatments, always dissatisfied with previous results. They adopt compulsive ritualistic behaviors such as looking in mirrors and using makeup or clothes that cover the body. They also overuse the prescribed treatments, such as exfoliating soaps and excessive use of acids.^7^ Severely affected individual avoid social life. It is estimated that up to 80% of individuals with BDD have suicidal ideation during their lifetime, and 24%−28% have attempted suicide.^1^, ^9^, ^10^, ^11^, ^12^

BDD is underdiagnosed in dermatology, and its psychosocial aspects have been receiving greater focus in recent decades.^13^, ^14^ However, the multifactorial aspects that lead to the onset of the disease are not yet fully understood, especially in Brazil.

The Body Dysmorphic Disorder Examination (BDDE) is a specific instrument for assessing BDD, developed in 1996 and translated into Brazilian Portuguese in 2008.^15^, ^16^, ^17^ It is a questionnaire with 34 items that assess the intensity of symptoms linked to concern with and negative assessment of appearance; self-awareness, discomfort, and feeling of being observed in public; excessive care for appearance; avoidance of social situations or activities in public, or even avoidance of physical contact; camouflage of appearance with clothing, makeup, or body posture; self-observation behavior, in the form of self-inspection, dressing up repeatedly, seeking reassurance, and comparing oneself to other people.

This study aimed to explore the prevalence and variables associated with BDD in female dermatological patients in a Brazilian public institution.

## Methods

This was a cross-sectional study involving adult women attended at the public dermatological service of UNESP (Botucatu, SP, Brazil). The research was approved by the ethics committee (No. 969.426) and all participants signed an informed consent form. Women in care at the psychiatric clinic or those with evident cognitive impairment were excluded.

There was no randomization of the inclusion of the participants, who were approached consecutively after medical care at dermatological outpatient clinics with esthetic complaints, in order to adequately represent this category and allow comparisons between the strata of the sample.

The participants underwent a standardized demographic survey regarding age, phototype, medical or psychiatric diagnosis, educational status, and family income, in addition to the BBDE questionnaire. The sum of the scores of the BDDE items varies from 0 to 168 points, with higher scores indicating greater dissatisfaction with body image. There is no accepted scale for categorizing patients; however, scores equal to or greater than 66 points indicate a high degree of dissatisfaction with appearance. Its internal consistency was assessed by Cronbach's alpha coefficient.

Dermatological complaints were classified as esthetic or non-esthetic by a qualified dermatologist (H.A.M).

BDD was defined as BDDE scores > 66, and assessed according to the behavior of demographic and psychological covariates using a logistic regression model. Only the covariables that maintained significance (p < 0.05) in the final model were maintained in the multivariate model. The size of the effect was estimated by the odds ratio (OR) of the regression and its 95% CI. For the multivariate analysis, the missing data (< 5% per variable) were estimated using the multiple imputation technique, with ten iterations.^18^

The sensitivity analysis of the results was performed from the perceptual map by multiple correspondence analysis.^19^

Sample design calculation was based on a pre-test with 100 participants. The prevalence of BDD, up to 15 covariates (including dummy variables) indicated that a minimum of 80 cases of BDD was needed to conduct the analysis, thus requiring up to 220 participants.^20^

Data were analyzed using the IBM SPSS v. 25 software. A p-value ≤ 0.05 was considered significant.

## Results

A total of 223 women were evaluated. Table 1 presents the main clinical and demographic data; approximately half of the sample was of productive age (30−50 years), and the majority identified themselves as white (84%). The high prevalence of psychological problems (domestic violence, bullying, sexual abuse) is noteworthy, and over one-third of the sample (38%) showed a high degree of dissatisfaction with their image. The prevalence of BDD was 48% among women with esthetic complaints and 30% among the others (p < 0.01).

**Table 1:** Clinical and demographic data of adult women treated at a public dermatology service (n = 223).

Variables	Values
Age (years)^a^	41.7 (11.9)
≤ 30^b^	56 (25)
31 − 50^b^	106 (48)
> 50^b^	57 (26)
Educational status^b^	
Elementary school	60 (27)
High school	81 (36)
College or university degree	81 (36)
Phenotype^b^	
White	188 (84)
Mixed-race	23 (10)
Black	10 (5)
Family income (R$)^b^	
< 1,000	51 (23)
1,000 − 3,000	116 (52)
> 3,000	55 (25)
Lives with partner^b^	125 (56)
Salaried work^b^	123 (55)
Esthetic complaint^b^	102 (46)
Domestic violence^b^	21 (9)
Sexual abuse^b^	16 (7)
Bullying^b^	52 (23)
Parental alcoholism^b^	47 (21)
Suicidal ideation^b^	65 (29)
Suicide attempt^b^	22 (10)
Psychotropic use^b^	49 (22)
BDDE score^a^	59 (32)
BDDE > 66^b^	85 (38)

a Mean (standard deviation).

b n (%).

There was no refusal to participate or difficulty in filling out the questionnaire, and the BDDE showed adequate internal consistency, with Cronbach's alpha coefficient = 0.90 (95% CI: 0.88 − 0.92).

The dermatological diseases sampled are listed in Table 2 and some of the reports referring to the disgust with appearance were transcribed, verbatim, in Table 3.

**Table 2:** Groups of dermatological diseases sampled in this study (n = 223).

Diagnosis	n	%
Photoaging	58	26.0%
Melasma/dyschromias	35	15.7%
Acne/folliculitis	30	13.5%
Eczema	21	9.4%
Psoriasis	18	8.0%
Others	12	5.4%
Alopecia	9	4.0%
Cutaneous lupus	8	3.6%
Milium	3	1.3%
Rosacea	3	1.3%
Vitiligo	3	1.3%
Skin cancer	2	0.9%
Epidermal cyst	2	0.9%
Pustular dermatosis of skin folds	2	0.9%
Leprosy	2	0.9%
Ringworm	2	0.9%
Itching	2	0.9%
Keloid	2	0.9%
Viral wart	2	0.9%
Vibex	2	0.9%
Pseudoacanthosis nigricans	1	0.4%
Melanocytic nevus	1	0.4%
Urticaria	1	0.4%

**Table 3:** Transcript of the main reports regarding dissatisfaction with appearance in the last month.

Acne bothers me a lot; in general, I feel ugly.
My expression lines bother me a lot, I try to change my hair to disguise them.
These spots make me ugly and I have low self-esteem; I don't want to go out.
My hand bothers me, people look at it with disgust and they think it is contagious.
Before there was no spot, and now there is this horrible one on my face.
The lesions appear on the face, they are ugly and everyone stares.
The spots make me look older, I put on makeup and it doesn't look good.
The discoloration bothers me a lot, it gives the impression of being dirty, people keep asking what it is.
The spots on my legs won't allow me to wear different kinds of clothes.
The spots on my face bother me a lot, I've already lost jobs because of them.
The spots on my face bother me, when I take a photo it looks even uglier.
The drooping eyelids make me look like an older woman.
People notice my melasma.
Wrinkles and blemishes really bother me, I can't look in the mirror.
They keep looking at the spots on my face
I have many wrinkles, my skin is ugly.
Striae are the worst possible thing, a blow to self-esteem.
I think this mole is horrible, when you go somewhere everyone looks.
I don't like what I see on my face. The spots are very uncomfortable.
I don't want people to see my hands.
I have expression lines around my mouth, I feel terrible
I have to wear clothes to cover lupus lesions, I suffer a lot.
I'm ashamed of my varicose veins, I can't wear the clothes I want.
I avoid leaving the house as much as possible. Whenever I look at myself in the mirror, I only see the spots and keloids.
I used to be beautiful, but now I'm old and I feel ridiculous.
I worry about the fat deposits under my eyes, the spots. I've had plastic surgery, but it's no use.
I feel strange with the blemishes.
I feel ugly, I have a lot of dark circles and white hair.
I feel uncomfortable and ugly with acne.
I feel very uncomfortable when I don't wear makeup, the skin on my face is horrible.
My face is very spotted, the skin looks very aged and lifeless.
My skin is aged, I want everything to come off and a new one to grow.
My hands are very old and ugly. I fight against it, but it doesn't get better. I'm spotted.
Nothing in my appearance pleases me, if I could I would change everything. I received a bad job evaluation because of my appearance.
I don't like the skin on my forehead and eyes, there's a hole in my forehead.
I don't like the blemishes and scars. I don't think I'm beautiful, I have low self-esteem.
I don't like the wrinkles on my neck and the spot on my skin. People say I'm crazy, but I want to fix it.
I don't like the wrinkles around my eyes, especially when I smile.
I don't like anything about my appearance.
I don't like anything about my appearance. Others have a way of fixing it, I don't. There is redness on my face, spots, and flaws in my hair.
I never leave home without makeup to cover up my blemishes.
My face gets very ugly, very spotted, it upsets me, I get nervous.
My face bothers me, it has blemishes and holes, people stare.
I want to be without that spot. I've suffered since I was a child, because they think it's dirty.
If I could avoid it, I would never look in the mirror.
If I had money, I would do all of the possible procedures. Aging is difficult: I got a divorce because of that, I can't get another partner because of that. Men don't like old women.
I know people think, "Why doesn't she try to improve her appearance?"
Whenever I'm talking, I feel like they're looking at my blemishes and not at me.
I feel like I have a hard time getting a job because of the blemishes. I hate to look in the mirror and see these spots.
I have psoriasis on my nails. It hurts a lot, it affects my self-esteem, and people are afraid to eat the food I make because they are afraid that the disease is contagious.
Everything bothers me. My hair doesn't grow, my face is blemished, I look horrible in the photos.

Table 4 presents the bivariate and multivariate analyses according to the prevalence of BDD. It is important to note that seeking care due to esthetic complaints (OR = 2.05; p = 0.02), lower income (OR = 2.97; p = 0.01), history of domestic violence (OR = 3.23; p = 0.04), and suicidal ideation (OR = 4.22; p < 0.01) were independently associated with a greater risk of presenting BDD.

**Table 4:** Assessment of the prevalence of body dysmorphic disorder in terms of demographic covariates and psychological damage (n = 223).

Variables	BDDE score	Bivariate analysis			Multivariate analysis^a^		
	Mean (standard deviation)	Odds ratio	95% CI	p-value	Odds ratio	95% CI	p-value^b^
Age (years)		1.02	0.99 − 1.04	0.09			
≤ 30	51 (29)						
31 − 50	65 (32)						
> 50	56 (34)						
Phenotype		2.57	0.70 − 9.43	0.15			
White	58 (32)						
Mixed-race	63 (29)						
Black	73 (33)						
Educational status		0.87	0.81 − 0.94	< 0.01			
Elementary school	65 (32)						
High school	63 (32)						
College or university degree	51 (31)						
Esthetic complaint							
Yes	64 (33)	2.18	1.26 − 3.78	< 0.01	2.05	1.10 − 3.82	0.02
No	55 (31)						
Income (R$)							
< 1.000	67 (34)	3.82	1.71 − 8.52	< 0.01	2.97	1.27 − 6.99	0.01
1,000 − 3,000	63 (32)						
> 3,000	45 (26)						
Salaried work							
yes	59 (32)	0.88	0.51 − 1.52	0.65			
No	59 (33)						
Lives with partner							
Yes	61 (33)	1.00	0.58 − 1.72	0.99			
No	56 (31)						
Domestic violence							
Yes	84 (33)	6.17	2.17 − 17.55	< 0.01	3.23	1.02 − 10.23	0.04
No	57 (31)						
Sexual abuse							
Yes	78 (28)	3.95	1.32 − 11.82	0.01			
No	58 (32)						
Bullying							
Yes	70 (34)	1.71	0.91 − 3.21	0.09			
No	56 (31)						
Parental alcoholism							
Yes	68 (36)	2.20	1.14 − 4.22	0.02			
No	57 (31)						
Suicidal ideation							
Yes	77 (34)	5.40	2.89 − 10.07	< 0.01	4.22	2.15 − 8.29	< 0.01
No	52 (28)						
Use of psychotropics							
Yes	65 (36)	2.09	1.11 − 3.96	0.02			
No	57 (31)						

BDDE, body dysmorphic disorder examination.

R (Nagerlkerke = 0.29); Hosmer-Lemeshow test (p = 0.96).

a p (model) < 0.01; p (constant) < 0.01.

b adjusted p-value.

**Figure 1:**
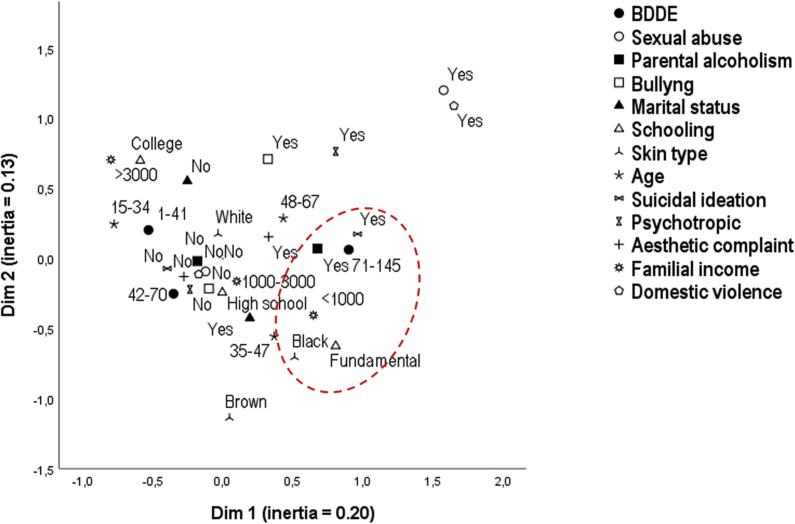
Perceptual map of the multiple correspondence analysis of the study variables (n = 223).

The perceptual map (multiple correspondence analysis) of the variables explained 33% of the variance of the complete model and is shown in Fig. 1, confirming the multidimensional proximity of the highest tertile of the BDDE scores with low education, african descent phenotype, parental alcoholism, lower income, and suicidal ideation. The multidimensional proximity of lowest tertile of BDDE scores with higher income, higher educational level, lower age group, and the denial of suicidal ideation and parental alcoholism was observed.

## Discussion

The skin is the main organ of social contact, and body appearance can represent concepts such as health, well-being, youth, fertility, and success. In a range of cultures, good-looking individuals are more likely to get better grades at school, to be hired for a job, to receive higher salaries, and to be seen as better, smarter, and healthier.^21^, ^22^ The search for improving appearance, maintaining youthfulness, raising self-esteem, and combating aging are demands of the collective, and represent a search for inclusion.

Medical care (plastic or esthetic) is part of the concept of health and disease, due to the damage to well-being that can be inflicted by the appearance. However, the perception of body image is deeply dependent on individual subjectivity, and can be influenced by cultural, economic, social, religious, and psychological experiences of each individual.^23^, ^24^

Self-image disorders greatly influence the search for skin and body care, which should alert medical professionals (especially plastic surgeons and dermatologists) to this demand, which has no correlation with the physical status of the condition.^13^, ^25^, ^26^ This study showed a high prevalence of excessive preoccupation with appearance among female dermatological patients, especially those with lower family income, personal history of domestic violence, and suicidal ideation, highlighting a psychological construction basis for BDD.

The evaluation of the participants' statements demonstrates the diversity of meanings that patients associate with body appearance: beauty, self-esteem, disgust, appearance of being older than their actual age, shame, calling attention, strangeness, rejection, failure. In the case of BDD, considering the behavioral strategies used, there is a predilection for escape or avoidance behaviors in relation to events in which their appearance can trigger aversive feelings, which promotes social isolation and favors the onset of affective disorders. Furthermore, patients with BDD have a frequent history of coercive educational practices, low social skills, great appreciation of appearance by people with whom they lived during childhood, and unpleasant events related to the part of the body with which they were concerned.^27^ Traumatic psychological episodes such as domestic violence, sexual abuse, bullying and parental alcoholism have repercussions on the construction of personality, including changes in self-esteem.^28^ The skin, as an organ of social contact, can induce stigmas linked to dermatological disease or esthetic complaints, which may be disproportionate to common sense.^29^

A controlled study with magnetic resonance imaging in eight women with BDD revealed morphometric changes in the caudate nucleus and a higher volume of white matter, similarly to what happens in obsessive compulsive disorder.^30^ In fact, in the psychiatric sphere, BDD is associated with obsessive-compulsive symptoms, although it is also associated with affective complaints (*e.g*., depression) and anxiety.^4^, ^9^, ^28^, ^31^

The study has limitations related to sampling in a public institution and the lack of randomization of the sample; however, the high frequency of esthetic complaints by the sampled patients may allow a certain inference for private dermatological activity, where this practice is more usual. Likewise, there was no obstacle to the internal comparative analysis of the subgroups.

Dermatologists should be aware of the high prevalence of BDD, especially in adult women with esthetic complaints, who present affective disorders or use of psychotropic drugs. The implemented procedures and treatments are unlikely to achieve satisfaction. This is primarily because skin complaints can result from the somatization of internal conflicts, and reveal a desire for change or permanent personal dissatisfaction, which can indicate psychological problems and result in suicidal ideation.^32^ It is essential to recognize the diagnosis in order to treat such patients and refer them to the appropriate psychologic or psychiatric treatment instead of trying to satisfy their esthetic demands.^33^

In dermatology, BDD is not only linked to dissatisfaction with treatments, but also to the urgency of its results (treatment overdose) and intolerance to adverse effects. The increase in longevity and the availability of technologies for skin and body care result in greater demand for esthetic care by the population. Careful selection of patients and procedures should lead to better levels of satisfaction and well-being.

## Conclusions

BDD is prevalent among female dermatological patients; it is associated with traumatic psychological experiences, lower income, affective disorders, and demand for esthetic care. It is essential to recognize the diagnosis in order to treat such patients and refer them to the appropriate psychologic or psychiatric treatment instead of trying to satisfy their demands.

## Financial support

FAPESP (no 2015/04592-5).

## Authors’ contributions

Mariana Mathias Morita: Approval of the final version of the manuscript; drafting and editing of the manuscript; collection, analysis, and interpretation of data; critical review of the literature; critical review of the manuscript.

Maira Renata Merlotto: Approval of the final version of the manuscript; drafting and editing of the manuscript; collection, analysis, and interpretation of data, intellectual participation in propaedeutic and/or therapeutic conduct of studied cases; critical review of the literature; critical review of the manuscript.

Cássia Lopes Dantas: Approval of the final version of the manuscript; design and planning of the study; collection, analysis, and interpretation of data; intellectual participation in propaedeutic and/or therapeutic conduct of studied cases; critical review of the manuscript.

Fernando Henrique Olivetti: Drafting and editing of the manuscript; collection, analysis, and interpretation of data; critical review of the manuscript.

Hélio Amante Miot: Statistical analysis; approval of the final version of the manuscript; conception and planning of the study; elaboration and writing of the manuscript; obtaining, analyzing, and interpreting the data; effective participation in research orientation; intellectual participation in propaedeutic and/or therapeutic conduct of studied cases; critical review of the literature; critical review of the manuscript.

## Conflicts of interest

None declared.
